# Global scientometric analysis of *Capnocytophaga canimorsus* infections: Research trends, collaboration networks, and thematic evolution within a One Health framework (2019–2024)

**DOI:** 10.14202/vetworld.2026.1932-1942

**Published:** 2026-05-12

**Authors:** Fran Espinoza-Carhuancho, Miguel Cabanillas-Lazo, Juan Alvitez, Abigail Temoche, Cesar Mauricio-Vilchez, Josmel Pacheco-Mendoza, Frank Mayta-Tovalino

**Affiliations:** 1 Grupo de Bibliometría, Evaluación de Evidencia y Revisiones Sistemáticas (BEERS), Universidad Científica del Sur, Lima, Perú; 2 Grupo de Investigación Neurociencias, Metabolismo, Efectividad Clínica y Sanitaria (NEMECS), Universidad Científica del Sur, Lima, Perú; 3 Unidad de Investigación, Innovación y Emprendimiento, Universidad Nacional Federico Villarreal, Lima, Perú; 4 Vicerrectorado de Investigación, Universidad San Ignacio de Loyola, Lima, Perú

**Keywords:** bacteremia, bibliometrics, Capnocytophaga canimorsus, dog bites, meningitis, One Health, scientometrics, zoonosis

## Abstract

**Background and Aim:**

*Capnocytophaga canimorsus* is an emerging zoonotic pathogen associated with severe infections, such as sepsis, meningitis, and endocarditis following animal bites. Despite increasing clinical recognition, a comprehensive understanding of global research trends and collaborative patterns remains limited. This study aimed to perform a scientometric analysis of global scientific output on *C. canimorsus* infections from 2019 to 2024, focusing on research trends, collaboration networks, and thematic evolution within a One Health framework.

**Materials and Methods:**

A descriptive observational scientometric study was conducted using the Scopus database. A structured search strategy identified 104 relevant documents published between 2019 and 2024. Bibliometric indicators, including publication output, citation metrics, and author h-index, were analyzed using SciVal and Bibliometrix in R. Keyword co-occurrence, thematic evolution, and international collaboration networks were evaluated to map research dynamics.

**Results:**

A total of 104 documents from 70 sources were analyzed, showing a slight negative annual growth rate (−5.59%). The mean document age was 2.64 years, with an average of 3.17 citations per publication. Research output involved 503 authors and 223 keywords, reflecting moderate thematic diversity but limited international collaboration (6.73%). Original articles predominated (n = 80), followed by reviews and other document types. Publication activity peaked in 2020 and 2022, with most studies appearing in mid-tier journals. Thematic analysis revealed a transition from early focus on infections and dog bites toward meningitis, bacteremia, and zoonotic diseases. Keyword clustering highlighted “meningitis,” “bacteremia,” and “dog bites” as central research themes. Spain and Belgium emerged as leading contributors in international collaborations, although global networking remained limited and unevenly distributed. Research trends increasingly emphasized zoonotic aspects of *C. canimorsus* within a One Health context.

**Conclusion:**

This scientometric analysis highlights evolving research priorities in *C. canimorsus* infections, with increasing emphasis on zoonosis and One Health perspectives. Despite growing scientific interest, limited international collaboration and underrepresentation of low-resource regions persist. Strengthening interdisciplinary and cross-regional partnerships is essential to enhance surveillance, diagnosis, and prevention strategies for this clinically significant zoonotic pathogen.

## INTRODUCTION

*Capnocytophaga canimorsus* is a Gram-negative bacterium that inhabits the oral cavities of dogs and cats and has gained attention as a zoonotic pathogen in human medicine [1, 2]. Although it rarely causes infections, *C. canimorsus* is a significant public health concern because it can induce severe, life-threatening diseases, particularly after animal bites or scratches [3]. *C. canimorsus* infection has approximately 0.67 case occurrence per media story about these infections (likely because *C. canimorsus* is infrequent compared to other bacterially based diseases). As noted above, most patients infected with *C. canimorsus* develop bacteremia (94%), with about one in three (35%) admitted to intensive care units for treatment of complications, such as endocarditis, septicemia, and disseminated intravascular coagulation [4].

Diagnosis of infection can be difficult, ranging from mild localized infections to fulminant sepsis, and often presenting with mild or vague symptoms that are difficult to detect clinically [4, 5]. Conventional culture is a less desirable method in many cases due to the time required for detection. Recent advancements in molecular diagnostic methods, such as polymerase chain reaction and real-time Nanopore sequencing, have shown promise in early detection, allowing more accurate and rapid identification of *C. canimorsus* in clinical settings [6, 7]. However, despite these technological advancements, the overall understanding of the epidemiology, pathogenesis, and optimal management of *C. canimorsus* infections remains limited [8].

There has been an increase in the number of scientific papers on *C. canimorsus* in recent years, reflecting a trend toward improved understanding of the organism and the management of infected patients. However, to our knowledge, there has not yet been a comprehensive bibliometric analysis of global research trends, key contributors, and collaboration networks in this area. Such analyses are useful for mapping the research landscape, identifying leading researchers and institutions, and highlighting emerging themes and gaps in the literature [9].

Earlier scientometric studies on *C. canimorsus* have addressed several aspects; however, case reports and clinical studies have received the majority of analytical attention. Genomic surveillance has been underexplored, and there is limited understanding of the human–veterinary interface or of global collaboration networks shaping research output. This thematic spread, where clinical infection is mapped to a zoonotic context as *C. canimorsus* emerges at the human–animal interface, does not adequately connect to One Health drivers. Future work extending beyond descriptive bibliometrics should incorporate interdisciplinary data to provide more rigorous insights into the behavior and impact of this pathogen [9].

Research on *C. canimorsus* is particularly relevant because it integrates environmental, animal, and human health components within the One Health framework. Increasing contact between humans and companion animals, especially dogs and cats, creates opportunities for cross-species transmission, while environmental factors influence pathogen persistence and exposure risk. Scientometric analyses have highlighted a disconnect between veterinary and human medical research, with most studies focusing on human clinical outcomes and limited inclusion of veterinary data. Identifying and addressing these gaps is essential for improving surveillance, diagnosis, and prevention of zoonotic diseases and for strengthening preparedness and response strategies [8, 9].

Despite increasing scientific interest in *C. canimorsus*, significant gaps remain in the global understanding of research dynamics and interdisciplinary integration. Existing studies have primarily focused on clinical case reports and descriptive analyses, with limited attention to large-scale scientometric evaluation of research productivity, collaboration networks, and thematic evolution. Furthermore, there is inadequate representation of genomic surveillance studies, minimal exploration of the human–animal–environment interface, and poor integration of One Health perspectives in the current literature. International collaboration patterns remain fragmented and geographically uneven, particularly with underrepresentation of low- and middle-income regions. In addition, there is a lack of comprehensive mapping of emerging research themes, which limits the ability to identify priority areas for future investigation. Addressing these gaps is essential to enhance the global understanding of *C. canimorsus* infections and to support evidence-based strategies for zoonotic disease control.

Therefore, this study aimed to perform a comprehensive scientometric analysis of global scientific output on *C. canimorsus* infections in human medicine from 2019 to 2024. Specifically, the study evaluated publication trends, key contributing authors and institutions, citation patterns, and international collaboration networks. In addition, it analyzed thematic evolution and keyword co-occurrence to identify emerging research priorities and knowledge gaps within a One Health framework. By integrating these components, the study sought to provide a systematic overview of the research landscape and inform future interdisciplinary and cross-regional research strategies to improve surveillance, diagnosis, and prevention of *C. canimorsus* infections.

## MATERIALS AND METHODS

### Ethical approval

This study did not require ethical approval because it relied solely on the collection and analysis of secondary data from publicly available scientific databases.

### Study period and location

The study was conducted using data retrieved from the Scopus database. Data collection was performed on October 8, 2024, and included publications from 2019 to 2024.

### Study design

This scientometric analysis was elaborated and reported according to the recommendations of the Reporting and Measurement of Items for Bibliometric or Scientometric Studies in Health Sciences (RAMIBS) [10]. Scientometric analysis is particularly appropriate for pathogens such as *C. canimorsus*, which have low global incidence but high clinical severity, because traditional epidemiological data may be limited. By mapping publication trends, collaboration networks, and thematic evolution, scientometric approaches help identify knowledge gaps, research priorities, and influential contributors in the field.

### Data source and search strategy

The search formula used was: TITLE-ABS-KEY(“Capnocytophaga canimorsus”) AND TITLE-ABS-KEY(“sepsis” OR “septic shock” OR “endocarditis” OR “meningitis” OR “abscesses” OR “cellulitis” OR “septic arthritis” OR “peritonitis” OR “gangrene” OR “acute renal failure” OR “pneumonia”), identifying a total of 104 relevant documents.

### Data extraction

A descriptive, observational study was conducted, employing a comprehensive search in the Scopus database using the search formula detailed above. The retrieved data covered the period from 2019 to 2024.

### Eligibility criteria and screening process

The inclusion criteria comprised peer-reviewed articles, reviews, case reports, and other scientific documents in English directly addressing *C. canimorsus* infections in human medicine between 2019 and 2024. Exclusion criteria included conference abstracts without full text, non-indexed sources, and publications unrelated to the study topic. To ensure data integrity, duplicate records retrieved from Scopus were systematically identified and removed based on DOI matching and metadata verification, thereby ensuring that each document was counted only once in the analysis.

### Scientometric indicators and operational definitions

For the scientometric analyses, the records retrieved from Scopus were exported in comma-separated values format to ensure standardized handling and compatibility across platforms. The database was uploaded to SciVal for classification based on bibliometric indicators, including scientific output, views, citations per publication, and author h-index. The file was then uploaded to Bibliometrix in R Studio version 4.3.2 (https://posit.co/download/ rstudio-desktop) for further analysis, where parameters were explicitly defined to improve reproducibility. The association strength clustering algorithm was used for keyword co-occurrence analysis, with a minimum keyword frequency of two occurrences per cluster to reduce noise and highlight thematic structures. Statistical and graphical packages within R Studio were used to generate visualizations of keyword networks, thematic evolution, and international collaboration.

### Software and analytical tools

Document selection and extraction were conducted after searching Scopus using a predefined search formula. Relevant documents were exported in comma-separated values format for processing. The dataset was initially analyzed in SciVal (https://www.scival.com/home) based on bibliometric indicators, including scholarly output, views, citations per publication, and author h-index. Subsequently, the dataset was analyzed in Bibliometrix within R Studio using statistical and visualization packages such as ggplot2, igraph, and base graphics to perform keyword co-occurrence mapping, thematic evolution analysis, and evaluation of international collaboration networks.

## RESULTS

### Overall publication characteristics

From 2019 to 2024, 104 documents were analyzed, originating from 70 different countries. An annual growth rate of −5.59% indicated a slight decline in scientific production on *C. canimorsus* infections. The documents had a mean age of 2.64 years and an average of 3.17 citations per paper, with a total of 1,836 references. These works involved 503 authors and 223 keywords, indicating thematic diversity but limited international collaboration (6.73%). Most papers (~4.95 co-authors per study) were multi-authored, while only three were single-authored. Document type distribution showed that original articles (n = 80) predominated, followed by letters and notes (~8) and reviews (n = 9), indicating that case-based papers dominate this research area (Table 1).

**Table 1:** Main characteristics of the analyzed publications.

Main description	Results
Timespan	2019–2024
Sources	70
Documents	104
Annual growth (%)	−5.59
Document average age (years)	2.64
Average citations per document	3.173
References	1836
Author’s keywords	223
Authors	503
Authors of single-authored documents	3
Single-authored documents	3
Co-authors per document	4.95
International co-authorships (%)	6.731
Article	80
Book chapter	1
Conference paper	1
Erratum	1
Letter	5
Note	6
Review	9
Short survey	1

The observed negative annual growth rate may be attributed to the rarity of reported cases of *C. canimorsus*, which limits opportunities for large-scale studies. Alternatively, this trend may reflect delays in indexing within databases such as Scopus, where recent publications may not yet be fully captured.

### Journal quartile distribution and publication trends

A total of 101 publications were distributed across CiteScore quartiles, demonstrating heterogeneous journal performance. Quartile 3 accounted for the majority (n = 39), followed by quartile 2 (n = 30) and quartile 1 (n = 21), while quartile 4 included only 11 publications. Year-wise analysis showed slight fluctuations, with peaks in 2020 and 2022 (20 and 21 publications, respectively) and a decline in 2024 (12 publications). Notably, quartile 3 journals dominated in 2022 (13 publications), suggesting a preference for mid-tier outlets during that period, whereas quartile 1 journals regained traction in 2024 with five publications. Overall, these findings highlight variability in journal impact and a tendency toward publication in mid-range sources (Table 2).

**Table 2:** Impact of scientific production according to quartile.

CiteScore quartile	Overall	2019	2020	2021	2022	2023	2024
Q1	21	4	3	2	4	3	5
Q2	30	4	6	6	3	7	4
Q3	39	5	8	4	13	6	3
Q4	11	2	3	5	1	0	0
Total	101	15	20	17	21	16	12

### Leading journals and citation impact

Analysis of Scopus-indexed sources revealed heterogeneity in journal impact. BMC Infectious Diseases had the highest number of publications (n = 8) and also showed a high CiteScore (6.5) with 5.6 citations per paper. BMJ Case Reports (n = 7) and IDCases (n = 6) had lower CiteScores (1.4 and 2.6, respectively), consistent with their focus on clinical case reporting rather than high-impact research. However, the highest citation impact was observed in the European Journal of Clinical Microbiology and Infectious Diseases, with 13.5 citations per paper and the highest CiteScore (10.4). Other journals, such as Antibiotics and APMIS, exhibited relatively high CiteScores but lower citation averages, indicating variability in visibility and scientific impact (Table 3).

**Table 3:** Top 10 scientific journals.

Scopus source	Scholarly output	CiteScore 2023	Citations per publication	SNIP 2023
BMC Infectious Diseases	8	6.5	5.6	1.11
BMJ Case Reports	7	1.4	4.3	0.34
IDCases	6	2.6	2.8	0.48
European Heart Journal – Case Reports	3	1.3	2.3	0.46
Infectious Diseases in Clinical Practice	3	0.8	0	0.1
American Journal of Emergency Medicine	2	6.0	2.5	0.95
Antibiotics	2	7.3	1	1.13
APMIS	2	5.2	0.5	0.76
Clinical Infection in Practice	2	2.1	0	0.4
European Journal of Clinical Microbiology and Infectious Diseases	2	10.4	13.5	1.1

SNIP = Source normalized impact per paper

### Author productivity and impact

Several authors contributed to research on *C. canimorsus*, particularly from Spain. Calvo-Muro, Del-Arco, and Fernández-Vecilla each published two papers with moderate citation averages (1.5) and h-index values of 2 and 3, respectively. Other contributors included Duployez and Loiëz, each with two publications and low citation impact (0.5), although Loiëz had a relatively high h-index (18). In contrast, Herget-Rosenthal and Rasmussen demonstrated higher impact, with citation averages of 15.5 and 3.5 and h-index values of 32 and 35, respectively, identifying them as leading contributors in this field (Table 4).

**Table 4:** Top 10 authors.

Author	Affiliation	Country/ Region	Scholarly output	Views count	Citations per publication	h-index
Calvo-Muro, Felicitas Elena	Hospital de Cruces	Spain	2	11	1.5	3
Del-Arco, José Luis Diaz De Tuesta	Hospital de Basurto	Spain	2	11	1.5	2
Duployez, Claire	Université de Lille	France	2	7	0.5	7
Fernández-Vecilla, Domingo	Hospital de Basurto	Spain	2	11	1.5	3
Herget-Rosenthal, Stefan	University of Duisburg-Essen	Germany	2	20	15.5	32
Langenbeck, Martin	Rotes-Kreuz-Krankenhaus	Germany	2	20	15.5	4
Loiëz, Caroline	Université de Lille	France	2	7	0.5	18
Lührs, Fabian	Rotes-Kreuz-Krankenhaus	Germany	2	20	15.5	2
Mader, Naomi	Rotes-Kreuz-Krankenhaus	Germany	2	20	15.5	2
Rasmussen, Magnus	Lund University	Sweden	2	19	3.5	35

### Core journal distribution based on Bradford’s law

**Figure 1:**
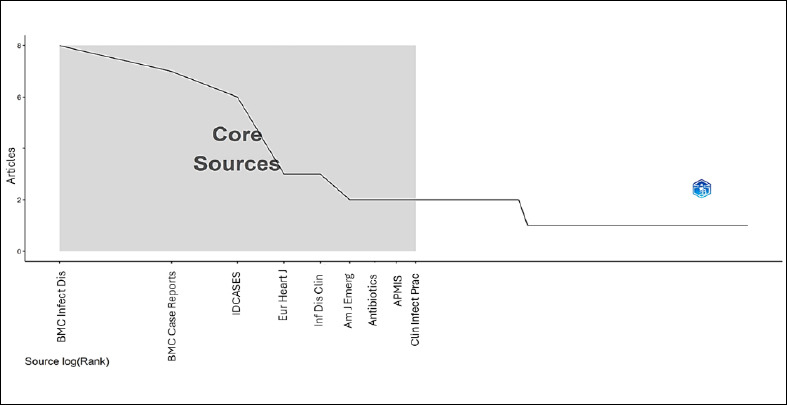
Main core sources.

The distribution of publications according to Bradford’s law identified core journals and zones of influence. Zone 1 included BMC Infectious Diseases (8 publications), BMJ Case Reports (7), and IDCases (6), representing the core sources where research is most concentrated. Zone 2 included the European Journal of Clinical Microbiology and Infectious Diseases and the European Review for Medical and Pharmacological Sciences, each with two publications. Zone 3 comprised sources such as Greene’s Infectious Diseases of the Dog and Cat, Fifth Edition, with one publication. In this classification, Zone 1 accounted for approximately one-third of total publications (n = 21), Zone 2 represented the second third (n = 20), and Zone 3 included the remaining sources (n = 19), reflecting the dispersion of scientific output across journals (Figure 1).

### Thematic evolution of research

**Figure 2:**
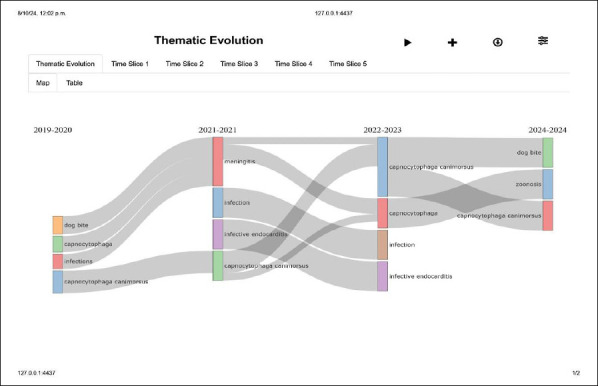
Thematic evolution.

Thematic evolution analysis showed a shift in research focus from *C. canimorsus* and meningitis between 2019 and 2024. Early studies primarily focused on infections and dog bites, gradually transitioning toward meningitis and bacteremia while maintaining consistent attention on *C. canimorsus*. From 2022 onward, research increasingly concentrated on zoonosis, highlighting evolving priorities in infectious disease research (Figure 2).

### Keyword co-occurrence patterns

**Figure 3:**
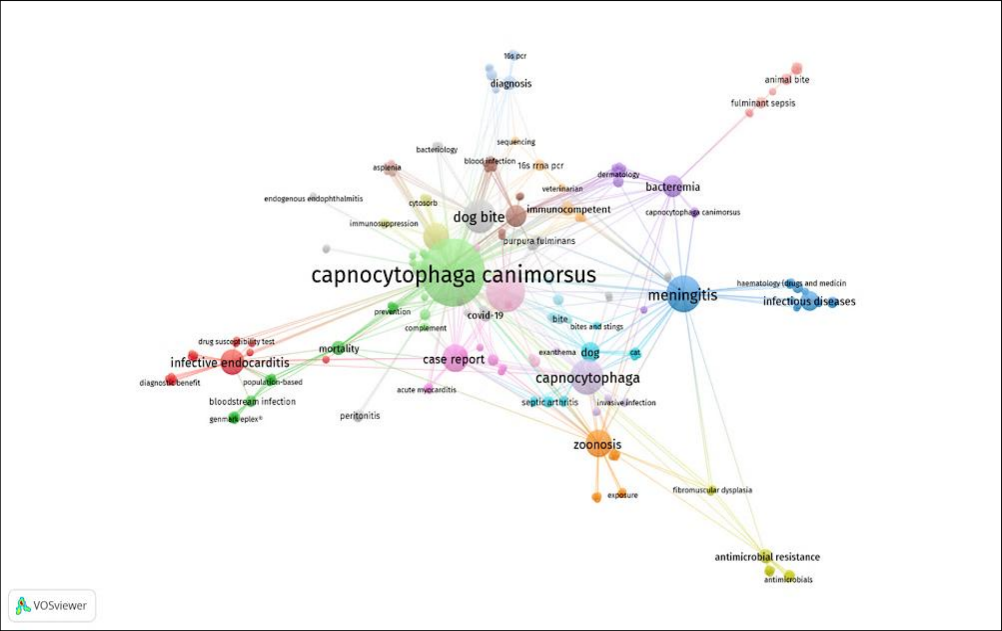
Co-occurrence per keyword.

Keyword co-occurrence analysis revealed multiple clusters, with the green cluster (*C. canimorsus*) representing the largest node, indicating that this term was central and highly interconnected with other research themes. The blue cluster (meningitis) and the red cluster (dog bite) were the next most prominent, reflecting their importance in the research landscape (Figure 3).

### International collaboration patterns

The cross-country collaboration map demonstrated several notable collaborations in recent studies. Spain and Belgium led with two collaborations. Additional collaborations were observed between Belgium and Haiti, Italy and Switzerland, and Qatar and Pakistan, each occurring once. Other collaborations included Sweden and Denmark, as well as the United States and Uganda, each at a frequency of one.

**Figure 4:**
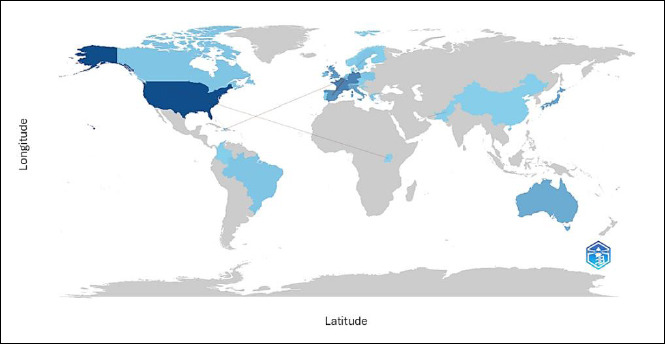
Country collaboration map.

These findings indicate geographic diversity in international research collaboration. When analyzed by continent, Europe accounted for the majority of collaborations, followed by North America, Asia, and Africa, with limited contributions. In contrast, Latin America and Oceania showed no scientific production or collaborations in this dataset, highlighting regional gaps. Furthermore, participation from countries such as Haiti, Uganda, and Pakistan indicates some involvement from low-income regions; however, these remain underrepresented compared to high-income countries, which dominate the collaborative landscape. This imbalance underscores the need to strengthen global research networks and promote the inclusion of resource-limited settings in zoonotic research (Figure 4).

## DISCUSSION

### Overview of study period and research trends

The study of the literature on *C. canimorsus* infections from 2019 to 2024 was purposefully selected to focus on the most recent scientific publications and to observe trends in research directions across borders. This period represents a phase of considerable dynamism in global science, characterized by growing awareness of zoonotic infections and by the COVID-19 pandemic’s influence on research and publication practices. Although case reports and scientific documentation of *C. canimorsus* infections have been available for several decades, the present study focused on the 2019–2024 period to capture more recent trends.

### Thematic evolution and One Health perspective

The gradual thematic shift from infections and dog bites toward zoonosis observed in these studies reflects broader One Health drivers at the global level. Similar to the impact of COVID-19 on infectious disease awareness, the increase in dog ownership worldwide, along with advancements in diagnostics and biotechnology, has contributed to re-framing subsequent studies of *C. canimorsus* within a zoonotic context.

Interpretation of strong collaborations, such as those between Spain and Belgium, should be approached with caution. While these countries possess established expertise and research infrastructure, their dominance also highlights the need for broader multinational collaboration. Zoonotic threats require coordinated global responses across diverse health systems, emphasizing the importance of expanding international research networks.

### Pathogen relevance and research importance

The species *C. canimorsus*, although less studied compared to other zoonotic pathogens, has gained increasing attention due to its role in severe human infections, particularly following animal bites or scratches. Environmental factors, including close contact between humans and domestic animals, combined with challenges in early diagnosis and treatment, have contributed to the growing interest in understanding this pathogen [3, 11].

### Role of bibliometric approaches

Bibliometric studies are widely used to assess characteristics of scientific literature within specific disciplines. Scopus, recognized for its comprehensive database of publications and citations, provides tools for analyzing authorship and citation patterns. Compared with other databases, such as Web of Science, Scopus includes a broader range of references and publications, offering a more extensive perspective [12]. Consequently, it has been widely used in previous bibliometric studies focusing on infectious diseases in human medicine [13, 14].

### Journal impact and publication quality

During the study period, the proportion of articles published in journals indexed in the first two quartiles was comparable to that in the lower quartiles. This suggests that novelty and scientific quality remain key determinants for publication in high-impact journals [15]. Furthermore, the absence of publications in the lowest quartile during the last two years may indicate increasing relevance and scientific value of recent research in this field.

Additionally, among journals with the highest number of publications, approximately half were classified in the first or second quartile, ensuring broader visibility and dissemination. High-quartile journals are more likely to reach a wider scientific audience and provide estimates closer to true values [16]. Therefore, careful journal selection is essential to maximize the impact and relevance of research findings, particularly in specialized fields such as infectious diseases and microbiology. Notably, the inclusion of a cardiology-related journal may be explained by the high mortality associated with *C. canimorsus* endocarditis, which has been reported to be approximately 25% [17].

### Authorship trends and citation patterns

Among the top 10 authors, all contributed two publications; however, four were co-authors of a systematic review of case reports that received 21 citations. This observation is consistent with evidence that systematic reviews tend to receive more citations than narrative reviews, even when published in the same journal [18]. Systematic reviews synthesize large volumes of evidence and are considered robust resources for clinicians and researchers.

### Evolution of research themes

Thematic evolution analysis demonstrated that early research primarily focused on case reports, particularly involving immunosuppressed patients following animal bites, meningitis, and superficial infections [19, 20]. In more recent years, the focus has shifted toward evidence synthesis and diagnostic studies. However, clustering patterns indicate that case report production remains ongoing. The growing global attention to zoonoses, particularly in the context of emerging infectious diseases, likely contributes to this thematic shift [21].

### International collaboration patterns

Spain emerged as the leading country in international collaboration in this field. This finding aligns with previous bibliometric analyses showing that a substantial proportion of Spanish research in infectious diseases involves international collaboration [22]. Such collaborations provide benefits, including shared expertise, access to funding, and increased opportunities for joint publications.

International collaborations also enable inclusion of diverse populations, enhancing generalizability and expanding the scope of infectious disease research [23–25]. Despite a high proportion of studies originating from the American and European regions, limited collaboration between them has been reported, highlighting the need to strengthen intercontinental research networks.

### Study limitations

Several limitations should be considered. First, the available literature on *C. canimorsus* remains limited, emphasizing the need for primary studies to generate stronger evidence. Second, reliance on a single database (Scopus) may have resulted in the omission of relevant publications indexed elsewhere. This may also contribute to the underrepresentation of non-English publications, potentially excluding research from regions where other languages are used.

Third, the automated clustering approach used in thematic evolution analysis may limit interpretation of causal relationships, as clusters are generated based on algorithmic parameters rather than conceptual linkages [24]. Finally, citation counts were used as a measure of impact; however, this metric inherently favors older publications that have had more time to accumulate citations, potentially biasing the interpretation of recent trends.

### Implications for One Health and future research

The identified scientometric trends in *C. canimorsus* research highlight the importance of a One Health perspective, as the progression from case reports to zoonotic studies reflects pathways of emerging infectious diseases. The low level of international collaboration and predominance of medical authorship indicate ongoing gaps in cross-disciplinary engagement, particularly between veterinary and human health sectors.

Expanding interdisciplinary and international research networks is essential to improve the detection, diagnosis, and prevention of zoonotic diseases. Integrating *C. canimorsus* research within the broader One Health framework will enhance understanding of drivers of zoonotic emergence, including pet ownership patterns and human–animal interactions. Future research should further explore these relationships to strengthen global preparedness and response strategies for zoonotic threats.

## CONCLUSION

This study provides a comprehensive scientometric overview of global research on *C. canimorsus* infections from 2019 to 2024, revealing important trends in publication output, thematic evolution, and collaboration patterns. The analysis demonstrated a slight decline in annual scientific production (−5.59%), with 104 documents originating from 70 countries and involving 503 authors. Research activity was predominantly characterized by case-based studies, with original articles accounting for the majority of publications. Journal distribution showed a concentration in mid-tier outlets, although high-impact journals contributed substantially to citation influence. Thematic analysis highlighted a transition from early emphasis on infections and dog bites toward meningitis, bacteremia, and, more recently, zoonosis within a One Health context. Despite these advances, international collaboration remained limited (6.73%) and unevenly distributed, with a strong dominance of European countries and underrepresentation of low-resource regions.

A major strength of this study lies in its systematic and reproducible scientometric approach, integrating bibliometric indicators, thematic mapping, and collaboration network analysis using standardized tools. The use of a well-defined search strategy and multiple analytical platforms enabled a robust evaluation of research productivity, impact, and emerging themes. In addition, applying a One Health perspective provides a broader interpretation of the findings, linking the human, animal, and environmental dimensions of *C. canimorsus* infections.

Future research should focus on strengthening global and interdisciplinary collaboration, particularly by increasing the participation of low- and middle-income countries. Greater emphasis on genomic surveillance, molecular epidemiology, and integrated One Health frameworks is needed to better understand transmission dynamics and disease burden. Furthermore, expanding research beyond case reports toward large-scale observational and experimental studies will enhance the evidence base for clinical management and prevention strategies. Inclusion of veterinary and environmental data will be critical to bridging existing knowledge gaps and improving cross-sectoral integration.

In conclusion, research on *C. canimorsus* is evolving toward a more integrated and zoonosis-focused framework; however, significant gaps remain in collaboration, data diversity, and interdisciplinary engagement. Addressing these limitations through coordinated global efforts and One Health-driven research strategies will be essential to improve surveillance, diagnosis, and prevention of this clinically significant zoonotic pathogen.

## DATA AVAILABILITY

The data generated during the study are fully included in the manuscript. Supplementary materials can be made available by the corresponding author upon reasonable request, ensuring transparency and reproducibility of the reported findings.

## AUTHORS’ CONTRIBUTIONS

FEC, JPM, FMT, CMV, MCL, JA, and AT: Conception of the study. FMT and FEC: Data extraction, verification, analysis, and writing – original draft and revision. All authors have read and approved the final version of the manuscript.
